# Artificial Intelligence for Diagnosis of Temporomandibular and Cranio-Cervico-Mandibular Musculoskeletal Disorders: A Systematic Review and Exploratory Diagnostic Test Accuracy Meta-Analysis

**DOI:** 10.3390/diagnostics16152468

**Published:** 2026-08-05

**Authors:** Arturo Arbeláez Ramírez, Daniel Botero Rosas

**Affiliations:** PhD Program in Bioscience, Faculty of Engineering, Universidad de La Sabana, Chía 250001, Cundinamarca, Colombia; arturoarra@unisabana.edu.co

**Keywords:** artificial intelligence, temporomandibular disorders, cranio-cervico-mandibular disorders, diagnostic accuracy, CBCT, MRI, DC/TMD

## Abstract

**Objectives**: To systematically evaluate the diagnostic accuracy, clinical applicability, and methodological maturity of artificial intelligence (AI)-based methods for temporomandibular disorders (TMD), temporomandibular joint (TMJ) abnormalities, and related cranio-cervico-mandibular (CCM) musculoskeletal conditions compared with conventional diagnostic methods and accepted reference standards. **Materials and Methods**: This systematic review and exploratory diagnostic test accuracy meta-analysis was conducted in accordance with PRISMA 2020 and PRISMA-DTA. The protocol was retrospectively registered in PROSPERO (CRD420261428138). PubMed/MEDLINE, Embase, and Scopus were searched from database inception through February 2026. Eligibility for the primary synthesis was restricted to published studies in English or Spanish involving adults aged 18 years or older. All extracted records were re-audited article by article to align the evidence with the diagnostic question. The domain-specific quantitative synthesis was restricted to TMJ osteoarthritis studies with explicit 2 × 2 diagnostic data or a unique, verifiable reconstruction from reported class totals and sensitivity/specificity. Risk of bias was assessed with QUADAS-2. **Results**: From 1471 records identified, 174 entered the master extraction dataset. After reclassification, 84 records were retained for primary TMD/TMJ qualitative synthesis, 8 as secondary CCM musculoskeletal evidence, 31 as conventional or reference standard supporting evidence, 33 as methodological or contextual evidence, 4 as differential orofacial pain evidence, and 14 as excluded or minimal-background records. Twenty-one studies were assessed as potential diagnostic accuracy candidates. Three TMJ osteoarthritis studies contributed to the domain-specific exploratory meta-analysis: two with explicit 2 × 2 data and one with a reproducible reconstruction. Pooled sensitivity was 0.791 (95% CI: 0.700–0.861) and pooled specificity was 0.869 (95% CI: 0.811–0.911). Heterogeneity was substantial for sensitivity (I^2^ = 68.2%) and moderate for specificity (I^2^ = 57.3%). **Conclusions**: AI demonstrates promising performance in selected image-based TMJ osteoarthritis tasks. Nevertheless, the evidence remains exploratory because only three studies were quantitatively comparable, one table was reconstructed, and modalities and validation designs differed. AI should be interpreted as an augmentative decision support tool rather than a replacement for MRI, CBCT, or validated clinical frameworks such as DC/TMD. Clinical Relevance: AI may support image-based TMD/TMJ workflows, but present evidence does not justify autonomous diagnosis or replacement of established clinical and imaging reference standards.

## 1. Introduction

Temporomandibular disorders (TMD) comprise a heterogeneous group of musculoskeletal and functional conditions involving the temporomandibular joints (TMJs), masticatory muscles, mandibular movement, and associated orofacial structures. They are among the most common causes of chronic orofacial pain and functional limitation. Population-based studies indicate that clinically significant TMD affects approximately 5–12% of adults, while signs or symptoms of dysfunction may be present in a substantially larger proportion of individuals. When cervical–mandibular interactions, upper cervical dysfunction, head posture, mandibular motor control, and masticatory muscle function are incorporated into the diagnostic framework, TMD overlaps clinically with the broader cranio-cervico-mandibular (CCM) musculoskeletal domain. This multidimensional character makes diagnosis challenging because similar symptoms may arise from intra-articular pathology, muscular pain, degenerative joint disease, disc displacement, altered mandibular dynamics, cervical dysfunction, sleep-related factors, or psychosocial amplification of pain [1,2,3,4,5].

Validated clinical frameworks remain central for diagnosis. The Diagnostic Criteria for Temporomandibular Disorders (DC/TMD) provide standardized criteria for pain-related and functional TMD subtypes and help distinguish muscular, articular, and headache-related entities. Imaging is complementary rather than universal. Magnetic resonance imaging (MRI) is the preferred reference method for disc position, joint effusion, inflammatory change, and soft-tissue assessment, whereas cone-beam computed tomography (CBCT) is the main structural modality for osseous degenerative change, cortical integrity, erosion, osteophytes, flattening, sclerosis, and condylar remodeling. However, structural findings may be present in asymptomatic individuals; therefore, imaging-based diagnosis must be interpreted within a clinical and functional framework rather than treated as an isolated gold standard [1,6,7,8,9,10,11].

Artificial intelligence (AI), including machine learning and deep learning, has increasingly been applied to dentomaxillofacial imaging and orofacial diagnostics. In TMJ research, AI models have been developed to detect osteoarthritis, classify degenerative joint disease, identify disc displacement, segment the articular disc or mandibular condyle, support radiographic interpretation, and integrate clinical or functional variables. These models may reduce observer variability, increase diagnostic efficiency, and identify subtle imaging patterns that are difficult to standardize visually. Nevertheless, high internal performance does not necessarily imply clinical readiness. Many studies use single-centre retrospective datasets, limited sample sizes, heterogeneous labels, incomplete external validation, and non-comparable outcome definitions [12,13,14,15,16,17,18,19,20,21].

Because diagnostic AI studies combine diagnostic accuracy design with complex imaging and data science workflows, transparent reporting is essential. The CLAIM 2024 update provides imaging-specific recommendations on dataset provenance, reference standards, model evaluation, internal and external testing, and reproducibility, whereas STARD-AI provides AI-specific diagnostic accuracy-reporting items addressing dataset practices, index test specification, algorithmic bias, fairness, applicability, and generalizability. Prospective adherence to these frameworks is necessary for reliable appraisal and future evidence synthesis [22,23].

Previous reviews have suggested that AI can achieve high diagnostic performance in selected TMD/TMJ tasks, but they have also emphasized low certainty of evidence, inconsistent reference standards, and incomplete reporting of diagnostic accuracy data. A major methodological problem is that diagnostic classification studies are frequently mixed with segmentation studies, prognostic models, risk prediction, broad orofacial pain applications, or general CCM-related AI work. These domains are clinically relevant, but they are not methodologically equivalent to diagnostic test accuracy (DTA) studies. Pooling them without strict classification can overestimate performance and generate misleading conclusions [24,25].

This review therefore re-evaluates the evidence using a corrected domain-specific framework. Studies were not excluded simply because they involved CCM, sleep, airway, headache, or orofacial pain; rather, they were classified according to their diagnostic relationship with TMD/TMJ, CCM musculoskeletal interaction, differential orofacial pain, conventional reference standards, or methodological context. This approach allows the primary synthesis to remain focused on AI for TMD/TMJ diagnosis while preserving clinically relevant CCM evidence for secondary interpretation. The objective was to evaluate the diagnostic accuracy and methodological maturity of AI-based approaches for TMD/TMJ and related CCM musculoskeletal disorders, while distinguishing primary DTA evidence from segmentation, prognostic, contextual, and differential-diagnosis evidence.

## 2. Materials and Methods

### 2.1. Study Design and Reporting Standards

**Figure 1:**
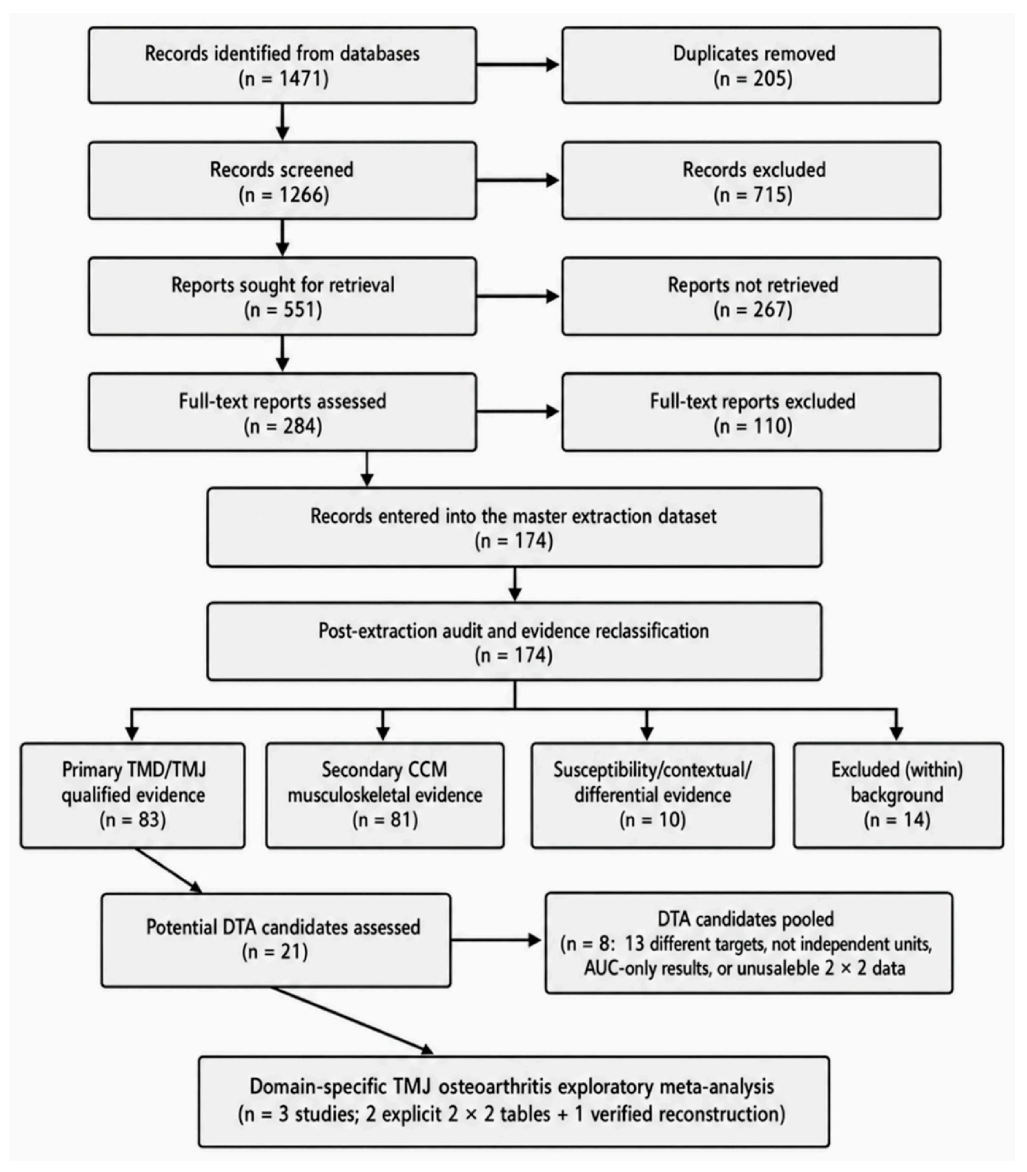
PRISMA 2020 flow diagram of the study selection process.

This systematic review and exploratory diagnostic test accuracy meta-analysis was conducted and reported in accordance with the Preferred Reporting Items for Systematic Reviews and Meta-Analyses 2020 statement (PRISMA 2020) and the PRISMA extension for Diagnostic Test Accuracy systematic reviews (PRISMA-DTA). The completed PRISMA 2020 and PRISMA-DTA checklists are provided as Supplementary Tables S1 and S2, respectively, and the study selection process is presented in Figure 1 [26,27,28].

The review was registered in the International Prospective Register of Systematic Reviews (PROSPERO; CRD420261428138; version 1.0 published on 22 June 2026; https://www.crd.york.ac.uk/PROSPERO/view/CRD420261428138 (accessed on 2 July 2026)). Registration occurred after the review had commenced and after the final database search; it is therefore retrospective. The review question, eligibility criteria, index tests, target conditions, reference standards, outcomes, and planned risk-of-bias assessment were specified in the protocol. The strict-versus-expanded evidence classification and the post-extraction audit were finalized during synthesis to prevent non-equivalent studies from being pooled. Because these analytical decisions were finalized after data extraction had begun, a potential risk of selective analytical decision-making cannot be excluded and is explicitly acknowledged in the Limitations section.

The review question was: What is the diagnostic accuracy of AI-based methods for detecting TMD, TMJ abnormalities, and related CCM musculoskeletal disorders compared with conventional diagnostic methods or reference standards such as MRI, CBCT, DC/TMD, expert radiological diagnosis, ultrasonography, electromyography, jaw tracking, or validated clinical protocols?

### 2.2. Eligibility Criteria

- Population: Published studies involving human adults aged 18 years or older, of any sex, with suspected or confirmed TMD, TMJ pathology, mandibular condylar abnormality, disc displacement, degenerative joint disease, masticatory muscle disorder, mandibular dysfunction, or a defensible CCM musculoskeletal condition. Animal, in vitro, cadaveric, simulation-only, pediatric, healthy-only, case report, editorial, narrative review, and conference abstract records were excluded from the primary diagnostic synthesis. Studies involving sleep, airway, headache, neuralgia, or facial pain were retained only as secondary or contextual evidence when they had a defensible relationship with mandibular, TMJ, or CCM function.
- Index test: AI-based diagnostic methods, including machine learning, deep learning, convolutional neural networks, radiomics, vision transformers, ensemble methods, automated image classification or detection, and multimodal diagnostic models. Segmentation-only studies were retained for qualitative synthesis but were not treated as diagnostic test accuracy studies unless they reported a prespecified diagnostic classification threshold.
- Comparator/reference standard: MRI for disc position and soft-tissue abnormalities; CBCT or CT for osseous abnormalities; DC/TMD or expert clinical diagnosis for clinically defined TMD; and, when appropriate to the target condition, expert radiological interpretation, ultrasonography, electromyography, jaw tracking, or another validated diagnostic protocol.
- Outcomes: Sensitivity, specificity, accuracy, area under the receiver operating characteristic curve (AUC), positive and negative likelihood ratios, diagnostic odds ratio, predictive values, and explicit or verifiably reconstructible 2 × 2 contingency table data. The analytical unit (patient, joint, image, or slice), diagnostic threshold, model, and validation dataset were recorded because these features determine whether estimates are independent and clinically comparable.

### 2.3. Search Strategy and Study Selection

PubMed/MEDLINE, Embase, and Scopus were searched from database inception through February 2026; the final documented PubMed search was run on 9 February 2026. Only published studies in English or Spanish were eligible, and no publication date restriction was applied. Search concepts covered artificial intelligence, machine learning, deep learning, TMD, TMJ, CCM disorders, orofacial pain, masticatory and cervical–mandibular function, MRI, CBCT, ultrasonography, electromyography, and diagnostic performance. The exact PubMed/MEDLINE strategy retained in the review archive is reproduced in Supplementary Table S3. The PROSPERO record confirms Embase and Scopus as the other information sources; however, the exact platform-specific archived strings were not present in the files available for this revision and are therefore not retrospectively reconstructed.

Two reviewers independently screened titles/abstracts and full texts against the predefined criteria. Disagreements were resolved by consensus. No automated eligibility decisions were used. After initial extraction, both reviewers re-audited the 174-record master dataset article by article to align each record with the diagnostic PICO and to prevent inappropriate pooling of non-comparable evidence. Study identification, screening, eligibility assessment, post-extraction classification, and quantitative inclusion are summarized In Figure 1. Authors of primary studies were not contacted for additional information, consistent with the PROSPERO record.

The diagram summarizes database identification, duplicate removal, title/abstract screening, report retrieval, full-text eligibility assessment, post-extraction audit, evidence classification, assessment of 21 potential DTA candidates, and inclusion of three domain-specific TMJ osteoarthritis studies in the exploratory meta-analysis. DTA, diagnostic test accuracy; TMD, temporomandibular disorders; TMJ, temporomandibular joint; CCM, cranio-cervico-mandibular.

### 2.4. Data Extraction and Post-Extraction Audit

Two reviewers independently extracted author, year, country, study design, sample size, target diagnosis, AI model, input modality, analytical unit, reference standard, validation strategy, diagnostic threshold, and performance metrics using a standardized form. For DTA candidates, TP, FN, FP, TN, sensitivity, specificity, and AUC were extracted when available. Disagreements were resolved by consensus. Missing information was recorded as not reported; it was not imputed except when a 2 × 2 table could be reproduced from an explicitly reported class denominator and sensitivity/specificity, as described below.

The post-extraction audit assigned each record to one mutually exclusive methodological role: primary TMD/TMJ diagnostic AI evidence, secondary CCM musculoskeletal evidence, differential orofacial pain evidence, conventional/reference standard evidence, dentomaxillofacial AI methodological context, review/contextual evidence, duplicate or non-independent evidence, or exclusion/minimal background. This classification allowed clinically relevant contextual evidence to be retained without treating it as equivalent to DTA evidence.

### 2.5. Diagnostic Accuracy Reconstruction Rules

Studies were prioritized for meta-analysis when they reported an explicit 2 × 2 table for a clinically relevant binary diagnosis. To address clinical heterogeneity, the principal expanded synthesis was restricted to the same target condition—TMJ osteoarthritis. A study was eligible for this domain-specific analysis only when disease-positive and disease-negative denominators and the corresponding sensitivity and specificity were explicitly reported, allowing a unique integer reconstruction of TP, FN, FP, and TN after rounding to the nearest count. The selected estimate had to represent a prespecified diagnostic model or threshold; multiple models, folds, mouth positions, or internal and external datasets from the same study were not entered as independent studies. Studies reporting only AUC, segmentation metrics, prognostic outcomes, non-independent cross-validation averages, a broader TMD-versus-healthy target, or multiple non-comparable image-level confusion matrices were not pooled. Complete derivations and reasons for non-pooling are reported in Supplementary Tables S4 and S5.

### 2.6. Risk of Bias and Applicability

Two reviewers independently assessed risk of bias and applicability with QUADAS-2 across patient selection, index test, reference standard, and flow/timing domains [29]. Disagreements were resolved by consensus. AI-specific concerns included data leakage, absence of blinding, post hoc threshold selection, inadequate separation of training and test data, non-independent cross-validation, lack of external validation, unclear class balance, and incomplete reporting of diagnostic contingency data.

### 2.7. Statistical Analysis

The conservative analysis included the two TMJ osteoarthritis studies with explicit 2 × 2 data. A domain-specific expanded exploratory analysis added [17], for which the reported class totals (100 TMJ-OA and 100 non-OA condyles) and sensitivity/specificity permitted a unique reconstruction. Sensitivity and specificity were pooled separately on the logit scale using random-effects models with DerSimonian–Laird between-study variance; a 0.5 continuity correction was prespecified for zero cells. Study-level 95% confidence intervals were calculated with the Wilson method, and statistical heterogeneity was evaluated using Cochran’s Q and I^2^. A bivariate or HSROC model would ordinarily be preferred because it accounts for the sensitivity–specificity correlation and threshold effects; however, with only three domain-specific studies, such models could not be estimated reliably. The separate pooled estimates are therefore presented as exploratory descriptive summaries rather than a universal diagnostic accuracy parameter. No subgroup analysis, meta-regression, formal certainty-of-evidence assessment, or Deeks test was performed. Analyses and figures were reproduced in Python 3.13 using NumPy 2.3.5, SciPy 1.17.0, statsmodels 0.14.6, and Matplotlib 3.10.8 [30,31].

### 2.8. Protocol Deviations

The database sources and eligibility details were harmonized with the final PROSPERO record. The protocol planned a bivariate random-effects model; because only three clinically comparable TMJ osteoarthritis studies had explicit or uniquely verifiable 2 × 2 data, the revision used separate random-effects logit models and reports this as a methodological deviation. The post-extraction audit was not a prespecified analytical stage; it was introduced after extraction to prevent segmentation, prognostic, CCM, and non-independent evidence from being pooled with DTA studies. The domain-specific restriction and verified reconstruction rules narrowed the synthesis without changing the diagnostic question. Registration remained retrospective, and these post hoc analytical decisions are explicitly treated as limitations.

## 3. Results

### 3.1. Study Selection and Corrected Evidence Classification

The database search identified 1471 records. After removal of 205 duplicates, 1266 records were screened by title and abstract, and 715 were excluded. Of 551 reports sought for retrieval, 267 were not retrieved and 284 full-text reports were assessed. A total of 110 full-text reports were excluded because they did not meet the target condition, index test, reference standard, study design, or extractable outcome requirements. The initial master extraction dataset contained 174 records. Because it included primary AI studies, conventional diagnostic studies, systematic reviews, methodological papers, CCM-related evidence, and differential orofacial pain articles, a post-extraction audit was completed before synthesis.

After article-by-article reclassification, 84 records were retained for primary TMD/TMJ qualitative synthesis, 8 for secondary CCM musculoskeletal interpretation, 31 as conventional/reference standard supporting evidence, 33 as methodological or contextual evidence, 4 as differential orofacial pain evidence, and 14 as excluded or minimal-background records. These mutually exclusive categories account for all 174 records and are summarized in Table 1.

### 3.2. Characteristics of the Qualitative Evidence

The primary TMD/TMJ evidence was dominated by imaging-based AI studies, especially CBCT, panoramic radiography, MRI, and cephalometric radiography. The main diagnostic targets were TMJ osteoarthritis or degenerative joint disease, anterior disc displacement, internal derangement, condylar morphology, articular disc segmentation, and mandibular functional analysis. Deep learning models, particularly convolutional neural networks and related architectures, were the most common methods for image-based tasks. Machine learning and ensemble approaches were more frequent in clinical-variable or functional datasets.

CCM-related studies contributed information on mandibular function, masticatory muscle activity, cervical–mandibular relationships, sleep/airway interactions, and broader musculoskeletal function. These studies were clinically relevant but were not pooled with the main TMJ/TMD diagnostic accuracy dataset unless the target condition, unit of analysis, and reference standard were comparable. The recommended use of each evidence category is summarized in Table 2, distinguishing primary diagnostic AI studies from segmentation, CCM, differential diagnosis, conventional reference standard, and contextual evidence.

### 3.3. Diagnostic Accuracy Candidates and Final Quantitative Dataset

Twenty-one studies were assessed as potential DTA candidates. Source-level verification identified two TMJ osteoarthritis studies with explicit 2 × 2 tables [15,16] and one TMJ osteoarthritis study with a uniquely reproducible reconstruction from reported class totals and sensitivity/specificity [17]. These three studies formed the domain-specific expanded exploratory synthesis. Haghnegahdar et al. 2018 evaluated a broader TMD-versus-healthy target using ten-fold cross-validation and was therefore not combined with TMJ osteoarthritis studies [18]. Fang et al. 2023 was not pooled because the accessible report emphasized AUC-based model validation without a verified threshold-specific 2 × 2 table [19]. Lin et al. 2022 reported five-fold image-level cross-validation averages rather than one independent 2 × 2 test set, and Yu et al. 2024 reported multiple internal/external and open-/closed-mouth image-level matrices that could not be represented by one independent comparable estimate [20,21]. These studies were retained as qualitative diagnostic evidence.

The quantitative synthesis therefore comprised three TMJ osteoarthritis studies: two explicit datasets and one verified reconstruction. The strict two-study analysis was retained as a conservative sensitivity analysis, whereas the three-study domain-specific analysis was treated as the principal exploratory estimate. No broad TMD classifier, image-level fold average, or unverified contingency table was used in the pooled result.

Table 3 presents the diagnostic target, analytical unit, validation design, verified contingency data, and quantitative role of each key DTA candidate. Supplementary Table S4 provides the arithmetic underlying the Nozawa reconstruction, and Supplementary Table S5 documents the reasons for retaining [18,19,20] outside the meta-analysis.

### 3.4. Pooled Diagnostic Performance

In the strict analysis of the two studies with explicit 2 × 2 data, pooled sensitivity was 0.748 (95% CI: 0.688–0.801; I^2^ = 0.0%) and pooled specificity was 0.864 (95% CI: 0.767–0.925; I^2^ = 77.6%). This analysis was methodologically conservative but statistically underpowered.

The domain-specific expanded analysis included three TMJ osteoarthritis studies: the two explicit datasets plus the verified reconstruction from [17]. Pooled sensitivity was 0.791 (95% CI: 0.700–0.861), with substantial heterogeneity (I^2^ = 68.2%), and pooled specificity was 0.869 (95% CI: 0.811–0.911), with moderate heterogeneity (I^2^ = 57.3%). These estimates are exploratory and summarize one diagnostic target across different imaging modalities and validation designs; they should not be interpreted as a universal accuracy estimate for all TMD or CCM conditions.

Refs. [18,19,20] were not entered into the pooled estimate because their available results did not provide one independent, clinically comparable, threshold-specific TMJ osteoarthritis 2 × 2 dataset. Consequently, the previously presented six- and seven-study pooled estimates were withdrawn. Formal small-study-effect or publication bias testing was not performed because only three studies were included.

The strict and domain-specific expanded pooled estimates are summarized in Table 4.

**Figure 2:**
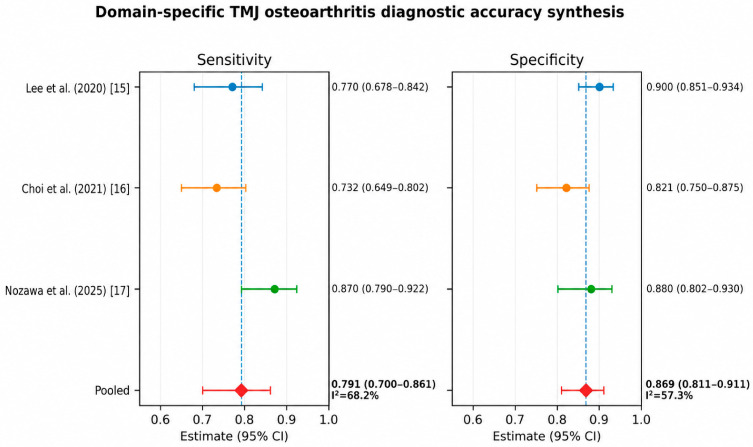
Forest plots of sensitivity and specificity for the domain-specific three-study TMJ osteoarthritis exploratory DTA synthesis [15,16,17].

The study-level sensitivity and specificity estimates for the domain-specific three-study synthesis are shown in Figure 2.

Study-level estimates are presented with Wilson 95% confidence intervals. Diamonds represent separate random-effects pooled estimates: sensitivity 0.791 (95% CI: 0.700–0.861) and specificity 0.869 (95% CI: 0.811–0.911).

**Figure 3:**
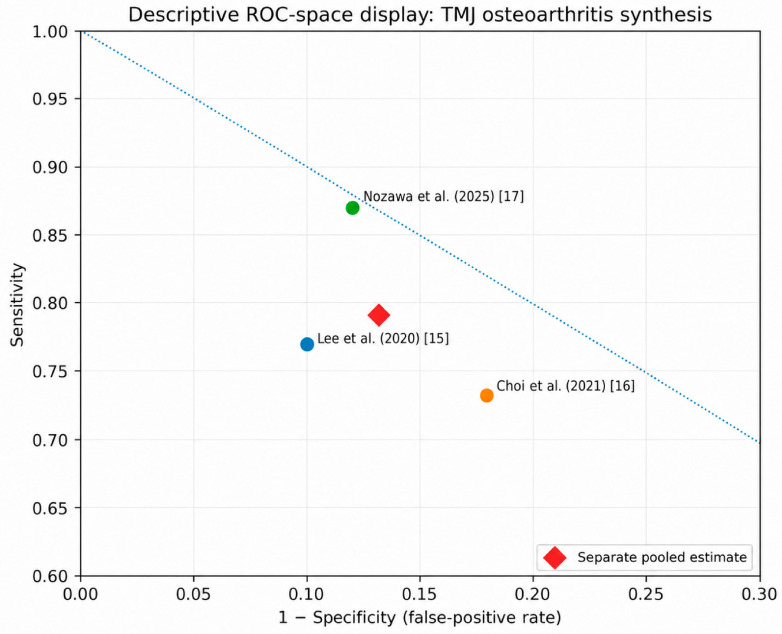
Descriptive ROC-space plot for the domain-specific three-study TMJ osteoarthritis exploratory synthesis [15,16,17].

Figure 3 displays individual study sensitivity against the false-positive rate and the separate pooled sensitivity/specificity estimate. It is descriptive and should not be interpreted as a fully estimated HSROC confidence or prediction region because only three studies were available.

### 3.5. Risk of Bias

**Figure 4:**
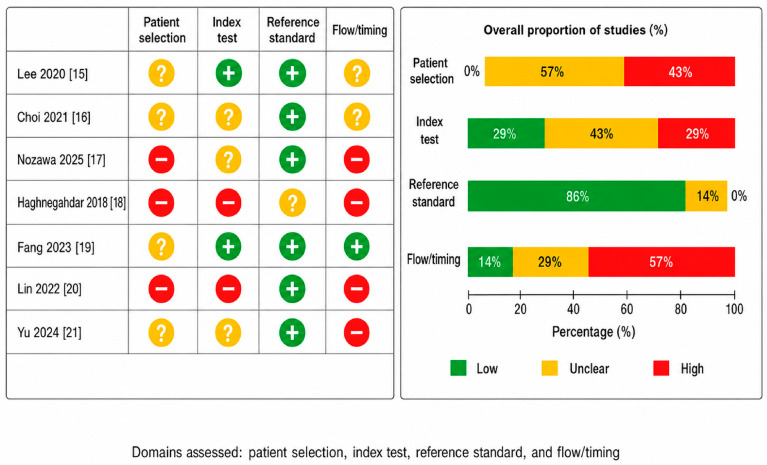
QUADAS-2 risk-of-bias judgments for the three studies in the domain-specific quantitative synthesis. “+” indicates low risk of bias, “?” indicates unclear risk of bias, and “−” indicates high risk of bias [15,16,17,18,19,20,21].

The QUADAS-2 assessment showed uneven methodological quality across the three studies in the domain-specific quantitative synthesis. The reference standard domain was generally the strongest. The main concerns were retrospective or convenience sampling, insufficient reporting of blinding and threshold specification, cross-validation or limited external validation, and unclear flow/timing. These features may overestimate performance and restrict clinical transportability. The principal domain-level concerns are summarized in Table 5, and study-level judgments are shown in Figure 4.

### 3.6. Comparative Interpretation Against Conventional and Reference Standards

AI performance was most convincing in image-based and anatomically defined diagnostic tasks. For osseous pathology, particularly TMJ osteoarthritis, AI-assisted CBCT, panoramic, and MRI models showed potential to standardize feature recognition. For soft-tissue diagnosis, MRI remained the reference standard for disc position and morphology, while AI was evaluated primarily as a classification or segmentation aid. For clinically defined pain disorders, including myalgia and arthralgia, DC/TMD remains the most established clinical framework, and AI has not demonstrated sufficient external validation to replace clinical examination. Ultrasonography, electromyography, and jaw movement analysis remain relevant functional or adjunctive methods, but their AI-enhanced diagnostic roles require further validation before they can be synthesized with TMJ imaging DTA evidence [32,33,34]. Table 6 summarizes the comparative interpretation.

## 4. Discussion

This systematic review indicates that AI has clinically relevant diagnostic potential in TMD/TMJ and related CCM musculoskeletal assessment, while also showing that the quantitatively comparable evidence base is smaller than suggested by broad AI performance summaries. The principal methodological correction was the separation of diagnostic classification studies from segmentation studies, prognostic models, contextual evidence, conventional reference standard studies, differential orofacial pain applications, and clinically different target conditions. This separation reduces the risk of pooling non-equivalent tasks.

The domain-specific three-study TMJ osteoarthritis exploratory synthesis yielded pooled sensitivity of 0.791 and specificity of 0.869. These values suggest promising performance for selected image-based TMJ osteoarthritis tasks; however, the small study count, modality differences, cross-validation in one study, and residual heterogeneity preclude interpretation as a universal accuracy estimate for all TMD or CCM conditions.

The distinction between the strict and domain-specific expanded analyses is important. The strict analysis included only two explicit 2 × 2 datasets and was therefore conservative but underpowered. The expanded analysis added only [17], for which the 100 TMJ-OA and 100 non-OA condyles and reported sensitivity/specificity permitted a reproducible integer reconstruction. [18] and the disc-displacement studies were not pooled because their target conditions, validation structures, or analytical units were not clinically comparable. This restriction directly addresses the risk of deriving a clinically ambiguous summary from heterogeneous disorders.

Compared with previous reviews, the present study provides a more conservative interpretation because it separates diagnostic classification studies from segmentation, prognostic, contextual, and broad orofacial-pain AI applications [22,23,24]. This distinction is important because pooled estimates may be inflated when heterogeneous AI tasks are treated as equivalent diagnostic accuracy evidence. The current review therefore shifts the interpretation from model performance alone toward methodological maturity, reproducibility, and clinical transportability.

The strongest evidence was found for structural imaging tasks. TMJ osteoarthritis and degenerative joint disease are suitable targets for AI because they involve definable radiographic signs such as erosion, flattening, osteophytes, sclerosis, cystic change, and condylar remodeling. AI systems may improve consistency in detecting these features, especially when expert dentomaxillofacial radiologists are not available. Nevertheless, AI models trained on CBCT, MRI, panoramic radiography, or expert-derived labels remain dependent on the quality of those labels and should not be considered independent gold standards.

For disc displacement, MRI remains the reference standard. Deep learning systems for detecting anterior disc displacement and segmenting the articular disc are promising, but current evidence is heterogeneous in terms of MRI sequences, diagnostic categories, unit of analysis, and validation design. Some studies report image-level or slice-level performance, while clinical diagnosis requires patient-level or joint-level interpretation. This distinction is critical because image-level datasets can inflate sample size and reduce the independence of observations.

The evidence for AI in clinically defined muscular, functional, and pain-related TMD remains less mature. TMD pain is multidimensional and includes biological, psychological, behavioral, and functional components. Models based only on imaging may fail to capture pain impact, disability, central sensitization, sleep disturbance, parafunction, and psychosocial factors. Therefore, AI systems for pain-related TMD should move toward multimodal integration, incorporating clinical examination, DC/TMD variables, patient-reported outcomes, functional measures, and imaging when clinically indicated.

The inclusion of CCM-related studies requires careful interpretation. Cranio-cervico-mandibular dysfunction is clinically relevant because mandibular movement, cervical posture, upper cervical mobility, masticatory muscle function, sleep, airway dimensions, and orofacial pain may interact. However, studies focused on sleep apnea, general airway analysis, headache, neuralgia, or broad musculoskeletal imaging should not be pooled with TMJ osteoarthritis or disc displacement unless the diagnostic target and reference standard are directly comparable. In this review, CCM-related studies were preserved as secondary evidence rather than excluded automatically, which provides a broader and more clinically nuanced interpretation without compromising the primary DTA analysis.

The most important limitation of the field is not the absence of high-performing AI models, but the lack of methodological standardization. Many studies used retrospective datasets, small or enriched samples, internal testing, inconsistent reference standards, and incomplete reporting of TP, FP, TN, and FN. Several studies reported AUC or accuracy but did not provide enough information to reconstruct diagnostic tables. This prevents robust meta-analysis and limits clinical translation. Future AI diagnostic studies in dentomaxillofacial radiology should prospectively follow STARD 2015, CLAIM 2024, and STARD-AI, provide complete contingency tables, prespecify thresholds, report calibration and clinically relevant units of analysis, and perform independent external testing [28,29,30].

From a clinical perspective, AI should currently be regarded as an augmentative diagnostic support technology. It may help prioritize cases, standardize interpretation, identify subtle radiographic features, and reduce observer variability. It should not replace expert radiological interpretation, MRI for soft-tissue assessment, CBCT for osseous evaluation, or DC/TMD-based clinical diagnosis. The most realistic near-term application is AI-assisted triage and decision support integrated into dentomaxillofacial imaging workflows.

This review has several strengths: a broad search strategy, explicit post-extraction audit of 174 records, separation of primary and secondary evidence domains, source-level reconciliation of DTA candidates, restriction of the meta-analysis to one target condition, and transparent distinction between explicit and reconstructed 2 × 2 data. The main limitations are the small number of meta-analysable studies, reliance on one reconstructed table, residual heterogeneity across modalities and validation designs, and inability to fit a reliable bivariate/HSROC model or perform subgroup analysis, meta-regression, certainty assessment, or publication bias testing. The exact archived Embase and Scopus search strings and the study-level log for the 110 reports excluded before formation of the master dataset were not available in the retained files; these gaps are reported transparently rather than reconstructed.

## 5. Conclusions

This review has several strengths: a broad diagnostic scope, an explicit post-extraction audit of 174 records, separation of primary and secondary evidence domains, source-level reconciliation of DTA candidates, and a domain-specific meta-analysis restricted to TMJ osteoarthritis. Its limitations are equally important. PROSPERO registration was retrospective, the post-extraction audit and strict-versus-expanded framework were finalized after extraction, only three studies contributed to the principal meta-analysis, one table required reconstruction, and validation designs differed. The small study count prevented reliable bivariate/HSROC modeling, subgroup analysis, meta-regression, formal certainty assessment, and Deeks testing.

Artificial intelligence shows promising diagnostic performance in selected TMD/TMJ applications, particularly anatomically defined image-based tasks such as TMJ osteoarthritis detection. In the domain-specific three-study exploratory synthesis, pooled sensitivity was 0.791 (95% CI: 0.700–0.861) and pooled specificity was 0.869 (95% CI: 0.811–0.911). These estimates should be interpreted cautiously because the evidence base is small, one contingency table was reconstructed, and imaging modalities and validation designs differed.

Current evidence does not support replacing established diagnostic standards. MRI remains essential for disc position and soft-tissue evaluation, CBCT remains central for osseous TMJ assessment, and DC/TMD remains the clinically validated framework for pain-related and functional TMD diagnosis. AI should therefore be positioned as an adjunctive decision support technology that may improve consistency, efficiency, and triage rather than as an autonomous diagnostic substitute.

Future research should prioritize prospective, multicentre, externally tested, and clinically integrated AI systems. Investigators should prospectively follow CLAIM 2024 and STARD-AI, report the analytical unit, prespecified thresholds, complete TP/FN/FP/TN tables, calibration, explainability, fairness, and independent external performance. Multimodal models should integrate imaging with clinical examination, function, and patient-reported outcomes. This transition is necessary before experimental model performance can be translated into reliable dentomaxillofacial diagnostic practice.

## Figures and Tables

**Table 1:** Corrected post-extraction classification of the 174-record master dataset. Records were classified according to their methodological role in the review rather than treated as equivalent diagnostic accuracy studies.

Category	Number of Records	Use in Manuscript
Primary TMD/TMJ/ATM AI evidence	84	Main qualitative synthesis
Secondary CCM musculoskeletal evidence	8	Secondary synthesis and clinical interpretation
Conventional/gold-standard or supporting evidence	31	Comparator framework, background, and interpretation
Methodological/contextual evidence	33	Introduction and discussion
Orofacial pain differential evidence	4	Differential diagnosis discussion
Excluded or minimal background	14	Not used in primary synthesis
Total	174	Master extraction dataset

**Table 2:** Recommended use of evidence categories. This table clarifies how broad TMD, CCM, orofacial pain, and methodological evidence should be handled to avoid inappropriate pooling.

Evidence Category	Examples	Recommended Use
Core TMD/TMJ diagnostic AI	TMJ-OA detection; disc displacement classification; TMD vs. healthy classification	Primary results and, when DTA data are available, meta-analysis
Segmentation or detection without diagnostic threshold	Disc segmentation; condyle segmentation; landmark detection	Qualitative synthesis only
CCM musculoskeletal evidence	Mandibular movement; masticatory muscle function; cervical–mandibular interaction	Secondary synthesis
Orofacial pain differential	Neuralgia; headache; facial pain classifiers	Discussion and differential diagnosis
Conventional reference standards	MRI; CBCT; DC/TMD; ultrasonography; EMG	Comparator and interpretation framework
Systematic reviews	Previous AI/TMD or TMJ-OA reviews	Background and comparison with prior literature

**Table 3:** Domain-specific diagnostic accuracy dataset and non-pooled key DTA candidates. Only the three TMJ osteoarthritis studies with explicit or reproducibly verified 2 × 2 data contributed to the exploratory meta-analysis.

Study	Target/Modality	Reference/Unit	Validation Design	TP/FN/FP/TN	Data Status	Quantitative Role
Lee et al. (2020) [15]	TMJ osteoarthritis/CBCT	Expert CBCT classification/image	Independent held-out test data	77/23/20/180	Explicit 2 × 2	Strict + domain-specific expanded
Choi et al. (2021) [16]	TMJ osteoarthritis/OPG	CBCT reference/image	Held-out Trial 3 test set	93/34/26/119	Explicit 2 × 2	Strict + domain-specific expanded
Nozawa et al. (2025) [17]	TMJ osteoarthritis/MRI PD-weighted	CT-supported radiological diagnosis/condyle	Five-fold cross-validation	87/13/12/88	Verified reconstruction	Domain-specific expanded
Haghnegahdar et al. (2018) [18]	TMD vs. healthy/CBCT texture	Clinical/radiological grouping/image	Ten-fold cross-validation	Not pooled	Different target and non-independent cross-validation	Qualitative only
Fang et al. (2023) [19]	Degenerative TMJ disease/cephalogram + clinical	Clinical/radiological diagnosis/patient	Training + validation cohorts	Not verified	AUC-focused; no verified threshold 2 × 2	Qualitative only
Lin et al. (2022) [20]	Anterior disc displacement/MRI	Expert MRI labels/image	Five-fold image-level cross-validation	Not independent	Fold-averaged metrics	Qualitative only
Yu et al. (2024) [21]	Anterior disc displacement/MRI	Expert MRI labels/image	Internal + external, open/closed-mouth sets	Multiple matrices	No single comparable estimate	Qualitative only

**Table 4:** Random-effects pooled diagnostic accuracy estimates. The three-study domain-specific TMJ osteoarthritis analysis is the principal exploratory result because every contributing table was explicit or reproducibly verified and the target condition was consistent.

Analysis	Studies	Pooled Sensitivity (95% CI)	Pooled Specificity (95% CI)	Heterogeneity	Interpretation
Strict explicit 2 × 2	2	0.748 (0.688–0.801)	0.864 (0.767–0.925)	Sensitivity I^2^ = 0.0%; specificity I^2^ = 77.6%	Conservative; underpowered
Domain-specific expanded exploratory	3	0.791 (0.700–0.861)	0.869 (0.811–0.911)	Sensitivity I^2^ = 68.2%; specificity I^2^ = 57.3%	Principal exploratory TMJ-OA estimate

**Table 5:** Summary of main QUADAS-2 concerns. The table summarizes the dominant sources of bias identified across AI diagnostic studies.

QUADAS-2 Domain	Main Concern	Likely Impact on Results
Patient selection	Retrospective, single-centre, enriched or convenience samples	May overestimate diagnostic performance and limit generalizability
Index test	Incomplete reporting of blinding, thresholds, training/test separation, and validation	Risk of data leakage and optimistic performance estimates
Reference standard	Variable labels: MRI, CBCT, expert opinion, clinical diagnosis, or mixed standards	Limits comparability across studies
Flow and timing	Unclear interval between index test and reference standard; incomplete patient flow	May introduce verification and timing bias
Applicability	Highly selected imaging datasets and limited external validation	Limits transferability to routine practice

**Table 6:** Comparative diagnostic interpretation of AI and conventional methods. AI should be interpreted as a diagnostic enhancer rather than a replacement for established reference standards.

Diagnostic Domain	Conventional/Reference Method	AI Role	Clinical Interpretation
TMJ osteoarthritis/osseous degeneration	CBCT or expert radiological interpretation	Automated detection/classification on CBCT, OPG, or MRI	Promising; strongest evidence domain
Disc displacement/internal derangement	MRI	Automated MRI classification and segmentation	Promising but dependent on MRI labels
Pain-related TMD/myalgia/arthralgia	DC/TMD clinical criteria	Clinical or multimodal prediction models	Evidence insufficient to replace DC/TMD
CCM musculoskeletal interaction	Clinical–functional assessment; imaging when indicated	Functional/multimodal AI models	Relevant for secondary synthesis; not yet mature for pooling
Orofacial pain differential diagnosis	Clinical differential diagnosis and specialist assessment	Decision support/rule-based or AI classifiers	Useful context but not equivalent to TMD/TMJ DTA

## Data Availability

The study-level quantitative dataset and reconstruction calculation used in the revised domain-specific meta-analysis are reported in Supplementary Table S4. The broader extraction dataset and post-extraction audit are available from the corresponding author upon reasonable request. The exact archived Embase and Scopus search strings and the study-level log for the 110 reports excluded before formation of the master dataset were not present in the retained review archive and were not retrospectively reconstructed.

## Supplementary Materials

The following supporting information can be downloaded at: https://www.mdpi.com/article/10.3390/diagnostics16152468/s1.
