# Supplementary material for: Artificial Intelligence for Diagnosis of Temporomandibular and Cranio-Cervico-Mandibular Musculoskeletal Disorders: A Systematic Review and Exploratory Diagnostic Test Accuracy Meta-Analysis
Source: Diagnostics (Basel). 2026 Aug 5;16(15):2468. doi: 10.3390/diagnostics16152468 (PMC13465469; doi:10.3390/diagnostics16152468)
Supplement: Supplementary file 1 [file diagnostics-16-02468-s001.zip › diagnostics-4450901-supplementary.pdf]

Supplementary material

Supplementary Table S1. PRISMA 2020 Checklist

The checklist reports the status of the revised manuscript. Items dependent on unavailable original search or early screening logs are identified transparently; no database syntax or exclusion reason was retrospectively invented.

Item	Section/topic	Checklist requirement (concise)	Location in manuscript	Reporting status in revised manuscript
1	Title	Identify the report as a systematic review.	Title page	Reported in Title page.
2	Abstract	Use a structured abstract reporting objectives, sources, eligibility, methods, results, limitations, registration and conclusions.	Abstract	Reported in the structured Abstract, including databases, registration, verified synthesis, limitations, and conclusions.
3	Rationale	Explain why the review was needed in the context of existing knowledge.	Introduction, paragraphs 1–4	Reported in Introduction, paragraphs 1–4.
4	Objectives	State the review objective/question explicitly.	End of Introduction; Section 2.1	Reported in End of Introduction; Section 2.1.
5	Eligibility criteria	Specify inclusion/exclusion criteria and how studies were grouped for synthesis.	Section 2.2	Reported in Section 2.2; adults ≥18 years, published English/Spanish studies, and evidence-grouping rules are specified.
6	Information sources	List all databases and other sources and date last searched.	Section 2.3; Supplementary Table S3	Reported in Section 2.3: PubMed/MEDLINE, Embase, and Scopus; searched through February 2026.
7	Search strategy	Provide complete search strategies for every database, including filters and limits.	Supplementary Table S3	Partially reported. PubMed/MEDLINE strategy is reproduced; exact archived Embase and Scopus platform strings were unavailable and were not retrospectively reconstructed.
8	Selection process	Describe number of reviewers, independence, tools/automation and disagreement resolution.	Section 2.3	Reported in Section 2.3: two independent reviewers; disagreements resolved by consensus.
9	Data collection process	Describe extraction procedures, number of reviewers, independence, and contact with authors.	Section 2.4	Reported in Section 2.4: two independent reviewers; authors were not contacted.
10a	Data items: outcomes	Define all outcomes sought and rules for selecting results.	Sections 2.2, 2.4, 2.5	Reported in Sections 2.2, 2.4, 2.5.
10b	Data items: other variables	Define study characteristics and assumptions for missing/unclear data.	Sections 2.4–2.5	Reported in Sections 2.4–2.5, including missing-data and reconstruction rules.
11	Risk of bias assessment	Describe tool, reviewers, independence, and any automation.	Section 2.6	Reported in Section 2.6: two independent assessors; consensus resolution.
12	Effect measures	Specify effect measures used for each outcome.	Section 2.7	Reported in Section 2.7.
13a	Synthesis: study eligibility	Describe how studies were judged eligible for each synthesis.	Sections 2.5 and 3.3	Reported in Sections 2.5 and 3.3.
13b	Synthesis: data preparation	Describe transformations, reconstruction, and handling of missing data.	Section 2.5	Reported in Section 2.5.

Item	Section/topic	Checklist requirement (concise)	Location in manuscript	Reporting status in revised manuscript
13c	Synthesis: presentation	Describe methods used to tabulate and visually display results.	Sections 2.7; Figures 2–4; Tables 3–5	Reported in Sections 2.7; Figures 2–4; Tables 3–5.
13d	Synthesis: statistical methods	Describe meta-analytic model, heterogeneity assessment, software and packages.	Section 2.7	Reported in Section 2.7, including model, heterogeneity methods, continuity correction, software, and versions.
13e	Synthesis: heterogeneity exploration	Describe subgroup, sensitivity or meta-regression methods.	Sections 2.7 and 3.4	Not performed because only four verified studies were available; stated in Sections 2.7 and 4.
13f	Synthesis: sensitivity analyses	Describe analyses assessing robustness.	Sections 2.5, 2.7, 3.4; Table 4	Reported in Sections 2.5, 2.7, 3.4; Table 4.
14	Reporting bias assessment	Describe assessment of missing results/small-study effects.	Section 2.7	Formal small-study-effect testing was not performed because only three studies were pooled.
15	Certainty assessment	Describe method used to assess certainty of evidence.	Methods/Discussion	Not formally assessed, as prespecified in PROSPERO; stated in Sections 2.7 and 4.
16a	Study selection results	Report selection from records to included studies, ideally with flow diagram.	Section 3.1; Figure 1	Reported in Section 3.1 and Figure 1.
16b	Excluded studies	Cite studies that appeared eligible but were excluded and explain why.	Section 3.1; Supplementary Table S5	Aggregate full-text exclusion counts are reported; key DTA exclusions and reasons are listed in Supplementary Table S5. The original study-level log for the 110 early full-text exclusions was unavailable.
17	Study characteristics	Cite included studies and present characteristics.	Sections 3.2–3.3; Tables 2–3	Reported in Sections 3.2–3.3; Tables 2–3.
18	Risk of bias results	Present risk-of-bias judgments for each included study.	Section 3.5; Figure 4; Table 5	Reported in Section 3.5; Figure 4; Table 5.
19	Results of individual studies	Present summary statistics and precision for each study.	Table 3; Figure 2	Reported in Table 3, Figure 2, and Supplementary Table S4.
20a	Synthesis results: contributing studies	Summarize characteristics and risk of bias of studies contributing to each synthesis.	Sections 3.3–3.5	Reported in Sections 3.3–3.5.
20b	Synthesis results: statistical summary	Report pooled estimates, confidence intervals and heterogeneity.	Section 3.4; Table 4; Figures 2–3	Reported for the strict two-study and domain-specific three-study exploratory analyses.
20c	Synthesis results: heterogeneity investigations	Report subgroup/meta-regression findings.	Section 3.4	Not performed because of the small and heterogeneous evidence base; stated in Sections 2.7 and 3.4.
20d	Synthesis results: sensitivity analyses	Report robustness analyses.	Section 3.4; Table 4; Table 4	Reported in Section 3.4; Table 4; Table 4.
21	Reporting biases	Present assessments of bias due to missing results.	Section 2.7; Discussion	Not assessed formally because only three studies were pooled.

Item	Section/topic	Checklist requirement (concise)	Location in manuscript	Reporting status in revised manuscript
22	Certainty of evidence	Present certainty/confidence judgments.	Discussion	Not formally assessed; stated as a limitation.
23a	Discussion: interpretation	Interpret results in relation to other evidence.	Discussion	Reported in Discussion.
23b	Discussion: evidence limitations	Discuss limitations of included evidence.	Discussion	Reported in Discussion.
23c	Discussion: review-process limitations	Discuss limitations of the review methods.	Discussion	Reported, including retrospective registration, post-extraction analytical decisions, and unavailable archived materials.
23d	Discussion: implications	Discuss implications for practice, policy and research.	Discussion and Conclusions	Reported in Discussion and Conclusions.
24a	Registration	Give registry name and registration number or state none.	Section 2.1	Reported in Section 2.1: PROSPERO CRD420261428138.
24b	Protocol	Indicate where the protocol can be accessed.	Section 2.1	Reported in Section 2.1 with the public PROSPERO record.
24c	Amendments	Describe and justify protocol amendments.	Section 2.1 or new Protocol Deviations subsection	Reported in Sections 2.8 and 4.
25	Support	Describe financial/nonfinancial support and sponsor role.	Funding	Reported in Funding.
26	Competing interests	Declare competing interests.	Declaration of competing interest	Reported in Declaration of competing interest.
27	Availability of data/materials/code	State availability of extraction forms, data, code and other materials.	Data availability	Reported in Data Availability and Supplementary Table S4.

Supplementary Table S2. PRISMA-DTA Checklist

This checklist applies PRISMA-DTA to the revised diagnostic accuracy review and distinguishes completed reporting from limitations caused by unavailable original search and early screening logs.

Item	Section/topic	Checklist requirement (concise)	Location in manuscript	Reporting status in revised manuscript
DTA-1	Title	Identify the report as a systematic review/meta-analysis of diagnostic test accuracy.	Title page	Reported in Title page.
DTA-2	Structured abstract	Include index test, target condition, reference standard, sources, accuracy results, limitations and registration.	Abstract	Reported in the structured Abstract.
DTA-3	Clinical background	Describe the clinical pathway, intended use and role of index tests.	Introduction; Section 3.6	Reported in Introduction; Section 3.6.
DTA-4	Review question	Frame objectives using participants, index test, target condition and reference standard.	Section 2.1	Reported in Section 2.1.
DTA-5	Eligibility criteria	Define study designs, participants, index tests, target conditions and reference standards.	Section 2.2	Reported in Section 2.2.

Item	Section/topic	Checklist requirement (concise)	Location in manuscript	Reporting status in revised manuscript
DTA-6	Information sources	List databases and last search date.	Section 2.3	Reported in Section 2.3: PubMed/MEDLINE, Embase, and Scopus through February 2026.
DTA-7	Search strategy	Provide reproducible strategies for all databases.	Supplementary Table S3	Partially reported: the retained PubMed/MEDLINE strategy is reproduced; archived Embase/Scopus strings were unavailable.
DTA-8	Study selection	Describe independent screening and disagreement resolution.	Section 2.3	Reported: two independent reviewers and consensus.
DTA-9	Data extraction	Describe duplicate extraction and extraction of 2×2 data, thresholds and indeterminate results.	Sections 2.4–2.5	Reported in Sections 2.4–2.5, including thresholds, units, missing data, and reconstruction.
DTA-10	Definitions	Define target condition, index test positivity, reference standard and unit of analysis.	Sections 2.2 and 2.5	Reported in Sections 2.2 and 2.5.
DTA-11	Risk of bias/applicability	Describe QUADAS-2 assessment and reviewer procedures.	Section 2.6	Reported in Section 2.6: two independent assessors and consensus.
DTA-12	Diagnostic accuracy measures	Specify sensitivity, specificity, likelihood ratios, DOR and ROC/SROC measures.	Sections 2.2 and 2.7	Reported in Sections 2.2 and 2.7.
DTA-13	Synthesis methods	Describe bivariate/HSROC or other model, continuity corrections, heterogeneity and software.	Section 2.7	Reported, including model, continuity correction, heterogeneity, software, and the reason a bivariate/HSROC model was not fitted reliably.
DTA-14	Multiple thresholds/tests	Explain how multiple thresholds, models or datasets from the same study were handled.	Section 2.5	Reported in Section 2.5; one estimate per independent study dataset was allowed.
DTA-15	Heterogeneity	Prespecify or describe sources of heterogeneity and subgroup/meta-regression plans.	Sections 2.7 and 3.4	The synthesis was restricted to the TMJ osteoarthritis domain; further subgroup/meta-regression was not feasible.
DTA-16	Sensitivity analyses	Describe strict and expanded analyses and unit-of-analysis decisions.	Sections 2.5, 2.7, 3.4	Reported in Sections 2.5, 2.7, 3.4.
DTA-17	Publication bias	Describe Deeks or other methods and minimum-study caveats.	Section 2.7	Deeks testing was not performed because only three studies were pooled.
DTA-18	Study selection results	Give numbers through screening and include flow diagram.	Section 3.1; Figure 1; Supplementary Table S5	Selection counts and aggregate reasons are reported; the original study-level log for the 110 early exclusions was unavailable.
DTA-19	Study characteristics	Present participant/sample characteristics, target conditions, index tests, reference standards and validation design.	Table 3; Section 3.2	Reported, including target, modality, unit, validation design, contingency data status, and quantitative role.
DTA-20	Individual accuracy results	Provide TP, FP, FN, TN and/or study-level sensitivity/specificity with CIs.	Table 3; Figure 2	Reported in Table 3, Figure 2, and Supplementary Table S4.
DTA-21	Risk of bias results	Present per-study QUADAS-2 judgments.	Figure 4; Table 5	Reported in Figure 4; Table 5.

Item	Section/topic	Checklist requirement (concise)	Location in manuscript	Reporting status in revised manuscript
DTA-22	Meta-analysis results	Report pooled sensitivity/specificity, confidence/prediction regions and heterogeneity.	Section 3.4; Table 4; Figures 2–3	Reported as separate random-effects pooled sensitivity and specificity for the domain-specific three-study synthesis; ROC-space display is descriptive.
DTA-23	Comparative accuracy	When comparing tests, describe direct/indirect comparisons and avoid overinterpretation.	Section 3.6	Narrative comparison only; no formal comparative meta-analysis was performed.
DTA-24	Additional analyses	Report subgroup, sensitivity and alternative-unit analyses.	Section 3.4; Table 4	Strict and verified expanded analyses are reported; image-level/non-independent studies were not pooled.
DTA-25	Discussion	Discuss accuracy, applicability, limitations, heterogeneity and clinical consequences.	Discussion	Reported in Discussion.
DTA-26	Conclusions	State implications without claiming replacement of accepted standards.	Conclusions	Reported in Conclusions.
DTA-27	Registration/protocol	Report registry, timing and protocol deviations.	Section 2.1	Reported in Sections 2.1, 2.8, and 4.

Supplementary Table S3 documents the retained PubMed/MEDLINE strategies and the information sources confirmed in PROSPERO.

Supplementary Table S3. Documented Search Strategies and Confirmed Information Sources.

Information source / search component	Documented strategy or status
**A. PubMed/MEDLINE AI-based search**	Search date: 9 February 2026. ("Artificial Intelligence"[MeSH] OR "AI" OR "Machine Learning"[MeSH] OR "Deep Learning"[MeSH] OR "Neural Networks, Computer"[MeSH] OR "Computer Vision" OR "Convolutional Neural Networks" OR "Vision Transformer" OR "ResNet" OR "DenseNet" OR "YOLO" OR "Xception" OR "Inception" OR "MobileNet" OR "VGG" OR "U-Net" OR "Radiomics" OR "Image Segmentation" OR "Segmentation" OR "Classification" OR "Detection" OR "Explainable AI" OR "XAI" OR "Expert Systems" OR "Robotics" OR "Supervised Learning" OR "Unsupervised Learning" OR "Knowledge-Based Systems" OR "Natural Language Processing") AND ("Temporomandibular Joint Disorders"[MeSH] OR "Temporomandibular Disorders" OR "TMJ Disorders" OR "Temporomandibular Joint Dysfunction Syndrome" OR "Craniomandibular Disorders" OR "Craniocervicomandibular Disorders" OR "Orofacial Pain"[MeSH] OR "Myofascial Pain Syndrome" OR "Facial Pain"[MeSH] OR "Neck Pain"[MeSH] OR "Cervicalgia" OR "Trigeminal Neuralgia"[MeSH] OR "Jaw Diseases"[MeSH] OR "Mandibular Diseases"[MeSH] OR "Masseter Muscle" OR "Temporalis Muscle" OR "Medial Pterygoid Muscle" OR "Lateral Pterygoid Muscle" OR "Cervical Muscles" OR "Suboccipital Muscles" OR "TMJ Capsule" OR "Temporomandibular Joint Disc" OR "Ligamentous Laxity" OR "Cartilage, Articular"[MeSH] OR "Joint Capsule"[MeSH] OR "Connective Tissue Diseases"[MeSH] OR "Temporomandibular Joint/blood supply"[MeSH] OR "Microcirculation" OR "Vascular Disorders"[MeSH] OR "Sleep Apnea, Obstructive"[MeSH] OR "Respiration Disorders" OR "Snoring" OR "Deglutition"[MeSH] OR "Swallowing Disorders"[MeSH] OR "Dysphagia" OR "Phonation" OR "Dysphonia" OR "Dysarthria" OR "Mandibular Function" OR "Jaw Movement" OR "Mastication"[MeSH] OR "Craniocervical Movement" OR "Cervical Range of Motion" OR "Neck Mobility" OR "Atlanto-Occipital Joint" OR "Upper Cervical Spine" OR "ATM" OR "CCM" OR "DTM" OR "Articulación Temporomandibular" OR "Disfunción temporomandibular" OR "Trastornos craneocervicomandibulares") AND ("Magnetic Resonance Imaging"[MeSH] OR "MRI" OR "Cone-Beam Computed Tomography"[MeSH] OR "CBCT" OR "Computed Tomography" OR "Radiography"[MeSH] OR "Panoramic Radiograph" OR "Cephalometry"[MeSH] OR "Electromyography"[MeSH] OR "EMG" OR "Ultrasound Imaging"[MeSH] OR "Ultrasonography"[MeSH] OR "Condylography" OR "Articulator" OR "Digital Articulator" OR "Kinesiography" OR "Jaw Tracking" OR "Motion Tracking Device" OR "Wearables" OR "DC-TMD" OR "Manual Palpation" OR "Clinical Examination") AND ("Sensitivity and Specificity"[MeSH] OR "ROC Curve"[MeSH] OR "True Positive Reactions" OR "False Negative Reactions" OR "Predictive Value of Tests"[MeSH] OR "Diagnostic Accuracy" OR "Diagnostic Performance" OR "Area Under Curve" OR "Likelihood Ratio" OR "Confusion Matrix" OR "F1 Score" OR "Precision" OR "Recall" OR "Dice coefficient" OR "Intersection over Union" OR "IoU")

B. PubMed/MEDLINE conventional-methods search	Search date: 9 February 2026. ("Temporomandibular Joint Disorders"[MeSH] OR "Temporomandibular Disorders" OR "Craniomandibular Disorders" OR "Craniocervicomandibular Disorders" OR "Orofacial Pain"[MeSH] OR "Myofascial Pain Syndrome" OR "Facial Pain"[MeSH] OR "Neck Pain"[MeSH] OR "Cervicalgia" OR "Trigeminal Neuralgia"[MeSH] OR "Jaw Diseases"[MeSH] OR "Mandibular Diseases"[MeSH]) AND ("Diagnosis"[MeSH] OR "Diagnostic Imaging"[MeSH] OR "Clinical Examination" OR "Diagnostic Accuracy" OR "Diagnostic Techniques" OR "Electromyography"[MeSH] OR "Condylography" OR "Jaw Tracking" OR "Kinesiography" OR "Palpation"[MeSH] OR "Auscultation"[MeSH] OR "MRI" OR "CBCT" OR "Ultrasound Imaging"[MeSH] OR "Cephalometry"[MeSH] OR "Panoramic Radiograph" OR "DC-TMD" OR "Thermography" OR "Vibration Analysis" OR "Acoustic Emissions") AND ("Sensitivity and Specificity"[MeSH] OR "ROC Curve"[MeSH] OR "Predictive Value of Tests"[MeSH] OR "Diagnostic Accuracy" OR "Diagnostic Performance" OR "Area Under Curve" OR "Likelihood Ratio" OR "True Positive Reactions" OR "False Negative Reactions" OR "Confusion Matrix") NOT ("Artificial Intelligence"[MeSH] OR "Machine Learning" OR "Deep Learning" OR "Neural Networks" OR "Computer-Aided Diagnosis")
C. Embase	Confirmed as an information source in PROSPERO CRD420261428138 and searched through February 2026. The exact archived platform-specific string was not present in the files available for this revision and is not retrospectively reconstructed.
D. Scopus	Confirmed as an information source in PROSPERO CRD420261428138 and searched through February 2026. The exact archived platform-specific string was not present in the files available for this revision and is not retrospectively reconstructed.
Restrictions	Published studies in English or Spanish; no publication-date restriction; final search period through February 2026.

Supplementary Table S4. Verified 2 × 2 Data and Reconstruction Calculation for the Domain-Specific TMJ Osteoarthritis Meta-Analysis.

Reconstructed counts were accepted only when the disease-positive and disease-negative denominators and the reported sensitivity and specificity produced one unique integer table. Nozawa et al. reported 100 TMJ-OA and 100 non-OA condyles; sensitivity 0.87 and specificity 0.88 therefore correspond to TP = 87, FN = 13, TN = 88, and FP = 12.

Study	D+	D−	TP	FN	FP	TN	Reported Se / Sp	Verification basis
Lee et al. 2020	100	200	77	23	20	180	0.77 / 0.90	Explicit table; combined held-out test data
Choi et al. 2021	127	145	93	34	26	119	0.73 / 0.82	Explicit held-out Trial 3 table
Nozawa et al. 2025	100	100	87	13	12	88	0.87 / 0.88	Unique reconstruction: 100 × 0.87 = 87 TP; 100 × 0.88 = 88 TN

Supplementary Table S5. Key Diagnostic Accuracy Candidates Retained Outside Quantitative Pooling.

Study	Diagnostic target	Reported design/output	Reason not pooled	Use in review
Haghnegahdar et al. 2018	TMD vs healthy	Ten-fold CBCT image cross-validation; sensitivity/specificity	Different target condition from TMJ osteoarthritis and no independent held-out test table	Qualitative diagnostic evidence
Fang et al. 2023	Degenerative TMJ disease	AUC-focused training/validation cohorts	No verified independent threshold-specific 2 × 2 table	Qualitative diagnostic evidence
Lin et al. 2022	Anterior disc displacement	Five-fold image-level cross-validation	Fold-averaged, non-independent results; different target condition	Qualitative diagnostic evidence
Yu et al. 2024	Anterior disc displacement	Multiple internal/external, open/closed-mouth matrices	No single independent comparable estimate; different target condition	Qualitative diagnostic evidence

Supplementary Figure S1. Broad seven-study exploratory sensitivity analysis including image-level cross-validation data.

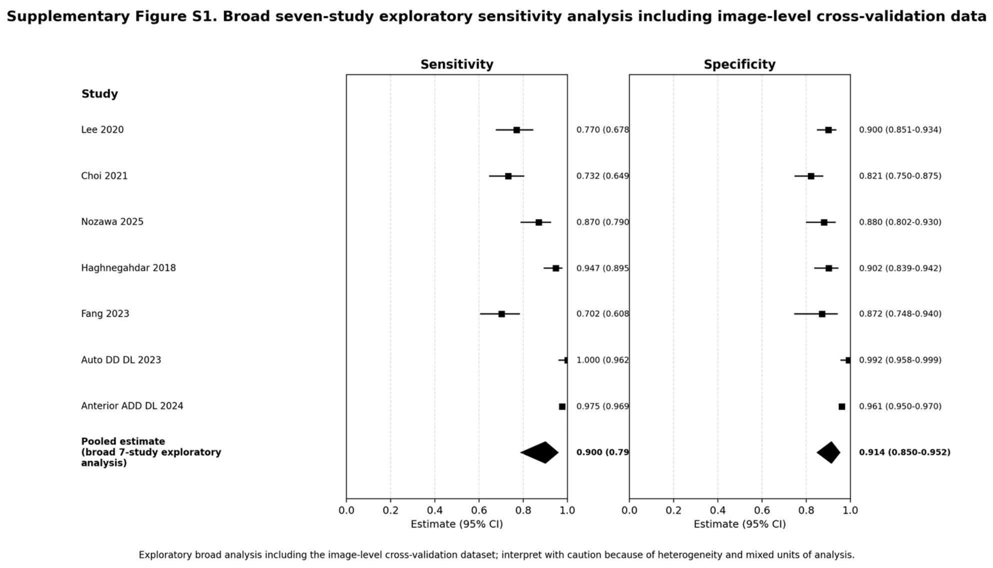

Exploratory broad analysis including the image-level cross-validation dataset; interpret with caution because of heterogeneity and mixed units of analysis
